# 

**Published:** 1874-08-15

**Authors:** 


No. XVI. CHICAGO, AUGUST 15, 1874. Vol. XV.

### THE Medical Examiner.

A

### Semi-Monthly Journal of Medical Sciences.

*TERMS.— Yearly Subscriptions, Three Dollars, in advance. Single Number Fifteen Cents. All Communications should be addressed to Publishers* MEDICAL EXAMINER, 65 *Randolph st., cor. State, Chicago.*

